# Metal‐based nanomaterials and nanocomposites as promising frontier in cancer chemotherapy

**DOI:** 10.1002/mco2.253

**Published:** 2023-04-04

**Authors:** Sunil Kumar, Monu Kumar Shukla, Abhishek Kumar Sharma, Gururaj K. Jayaprakash, Rajiv K. Tonk, Dinesh K. Chellappan, Sachin Kumar Singh, Kamal Dua, Faheem Ahmed, Sanjib Bhattacharyya, Deepak Kumar

**Affiliations:** ^1^ Department of Pharmaceutical Chemistry School of Pharmaceutical Sciences Shoolini University Solan Himachal Pradesh India; ^2^ School of Pharmaceutical Sciences Shoolini University Solan Himachal Pradesh India; ^3^ Department of Chemistry Nitte Meenakshi Institute of Technology Bangalore Karnataka India; ^4^ School of Pharmaceutical Sciences Delhi Pharmaceutical Sciences and Research University New Delhi Delhi India; ^5^ Department of Life Sciences International Medical University Kuala Lumpur Malaysia; ^6^ School of Pharmaceutical Sciences Lovely Professional University Phagwara Punjab India; ^7^ Discipline of Pharmacy, Graduate School of Health University of Technology Sydney Ultimo New South Wales Australia; ^8^ Discipline of Pharmacy, Graduate School of Health, University of Technology Sydney Sydney Australia; ^9^ Faculty of Health, Australian Research Centre in Complementary and Integrative Medicine University of Technology Sydney Sydney Australia; ^10^ Department of Physics College of Science King Faisal University Al‐Hofuf Al‐Ahsa Saudi Arabia; ^11^ Institute of Science Nirma University Ahmedabad Gujarat India

**Keywords:** cancer, chemotherapy, nanocomposites, nanomaterials, nanomedicine

## Abstract

Cancer is a disease associated with complex pathology and one of the most prevalent and leading reasons for mortality in the world. Current chemotherapy has challenges with cytotoxicity, selectivity, multidrug resistance, and the formation of stemlike cells. Nanomaterials (NMs) have unique properties that make them useful for various diagnostic and therapeutic purposes in cancer research. NMs can be engineered to target cancer cells for early detection and can deliver drugs directly to cancer cells, reducing side effects and improving treatment efficacy. Several of NMs can also be used for photothermal therapy to destroy cancer cells or enhance immune response to cancer by delivering immune‐stimulating molecules to immune cells or modulating the tumor microenvironment. NMs are being modified to overcome issues, such as toxicity, lack of selectivity, increase drug capacity, and bioavailability, for a wide spectrum of cancer therapies. To improve targeted drug delivery using nano‐carriers, noteworthy research is required. Several metal‐based NMs have been studied with the expectation of finding a cure for cancer treatment. In this review, the current development and the potential of plant and metal‐based NMs with their effects on size and shape have been discussed along with their more effective usage in cancer diagnosis and treatment.

## 1 INTRODUCTION

Cancer is a diseased condition that involves a complex pathophysiology and is the most prevalent and leading cause of mortality in the world.^1^ Nowadays, the screening and development of potential anticancer agents is an absolute necessity. In this regard, metal‐based nanomaterials (NMs) have potential to be antitumor agents, against a wide range of cancerous cells. NMs are defined as the materials with dimensions less than 100 nm.^2^ Several investigations have been conducted for the development of NMs in various shapes and sizes, such as nanotubes, nanoparticles (NPs), fibers, wires, and nanodots, depending upon the various diseases. NMs significantly improved the diagnosis and treatment of diseases via delivery systems, imaging, and photothermal agents due to their unique characteristics.^3^

Metal‐based NPs, such as gold and silver, have been frequently used in DNA hybridization detection, cell imaging, protein interaction, and photothermal therapy (PTT).^4^ The remarkable optical characteristics, accessible surface chemistry, and correct size scale enable silver and gold (AuNPs) highly promising for cancer diagnostic and therapeutic applications. Optimizing the size and shape of metal‐based NPs or conjugating them with certain ligands/biomarkers can enhance the cancer diagnosis and treatments.^2^

Drug delivery systems (DDSs) based on NMs have been shown to improve the therapeutic efficacy of anticancer treatments and minimize the side effects.^5^, ^6^ Several complications like inadequate tumor penetration capacity, inappropriate and nonspecific accumulation in tissues, early drug release into tissues, and unregulated drug release at the target location can be overcome depending upon the DDSs that were successfully investigated in human and animal models in both experimental and preclinical conditions.^7^

NPs based DDSs have considerable potential for the treatment of several types of cancer.^8^ The important technological advantages of NPs are they can be used as drug carriers with high carrier capacity, high stability, and feasibility in both hydrophobic and hydrophilic substances and can be used in various routes of administration, for example, oral application and inhalation route.^9^ NPS can also be designed to allow controlled drug release from the matrix. These properties of NPs enable the improvement of drug bioavailability and reduction of the dosing frequency and may resolve the problem of nonadherence to cancer therapy.^10^ Materials such as polymers, either natural (like gelatin or albumin) or synthetic (such as polylactides or poly(alkyl cyanoacrylates)), or solid lipids are used to make biocompatible and biodegradable NPs. Drugs contained in NPs are typically released from the matrix in the body through swelling, diffusion, erosion, or degradation.^11^ The field of cancer diagnosis and therapy has undergone various changes with the development of nanotechnology.^12^ The use of NPs (1–100 nm) in the treatment of cancer due to their drug‐like properties, such as stability, reduced toxicity, biocompatibility, enhanced permeability along with retention effect, and specificity.^13^ The NPs DDs is particular and utilizes tumor and tumor environment characteristics. NPs are able to overcome multidrug resistance in addition to the shortcomings of traditional cancer treatment.^14^ Several NMs are employed in cancer diagnosis given in Table 1.

**TABLE 1:** Nanomaterials (NMs) used in the diagnosis of cancer.

Nanomaterial	Application	Physical property	Detection medium	Conjugate	References
AuNPs	SNP detection	SPR blueshift with NP dispersion	Buffer	ssDNA	^16^
AuNPs and magnetic microparticles	PSA detection (prostate cancer)	Magnetic separation, high surface area	Buffer, serum	Antibody and ssDNA	^17^
AuNPs and magnetic microparticles	PSA detection (prostate cancer)	NP‐coated electrode surface area	Serum, cell lysate	Antibody	^18^
AuNPs and gold nanorods	PSA detection (prostate cancer)	Scattering	Buffer	Antibody	^19^
AuNPs	PSA detection (prostate cancer)	SPR blueshift with NP dispersion	Buffer	Designer peptides	^20^
AuNPs	PSA detection (prostate cancer)	SERS	Buffer	Antibody	^21^
AuNPs	PSA detection (prostate cancer)	SERS	Serum	Antibody	^21^
AuNPs	Protease detection	SPR blueshift with NP dispersion	Buffer	Designer peptides	^23^
AuNPs	Protease detection	SPR redshift with NP aggregation	Buffer	Designer peptides	^23^
AuNPs	Kinase detection	SPR redshift with NP aggregation	Cell lysate	Designer peptides	^25^
AuNPs	Kinase detection	SPR redshift with NP aggregation	Buffer	Designer peptides	^26^
AuNPs	Biomarker and DNA detection	Change in NP scattering and Brownian motion upon NP aggregation	Buffer, serum	Antibody, aptamers, and oligonucleotides	^26^
AuNPs	Biomarker and DNA detection	Fluorescence quenching	Buffer, cell culture medium	Aptamer (for PDGF detection)	^27^
AuNPs and magnetic microparticles	Biomarker and DNA detection	Magnetic separation, high surface area	Buffer	Antibody and ssDNA	^29^
AuNPs and magnetic microparticles	Biomarker and DNA detection	Magnetic separation, high surface area	Buffer, serum	Antibody and ssDNA	^30^
Cd‐free QDs	Assessment of the presence of cancerous cells in biopsied tissue	Photoluminescence	Cell culture medium	Anti‐claudin 4 and antiprostate stem cell antigen	^31^
Oval AuNPs	Assessment of the presence of cancerous cells in biopsied tissue	SPR redshift with NP aggregation	Buffer	Anti‐HER2 and aptamers	^32^
QD—AuNPs	Protease detection	FRET	Buffer	Designer peptides	^33^
QD‐coated magnetic microparticles	Assessment of the presence of cancerous cells in biopsied tissue	Magnetic separation and photoluminescence (multifunctional approach)	Cell culture medium	EGF	^34^
QDs	Kinase detection	FRET	Buffer	Designer peptides—dye‐labeled antibody	^35^
QDs	Protease detection	Bioluminescence resonance energy transfer	Buffer, serum	Designer peptide‐ bioluminescent protein	^36^
QDs	Protease detection	FRET	Buffer, cell culture medium	Designer peptide—dye	^35^
QDs	Protease detection	FRET	Buffer	Designer peptide—dye	^37^
QDs	PSA detection (prostate cancer)	Photoluminescence	Serum	Streptavidin	^38^
QDs	SNP detection	Photoluminescence	Buffer	ssDNA	^39^

Abbreviation: NP, nanoparticles.

There has been a lot of research done on different metallic NPs for biomedical uses.^40^ Due to their noteworthy inertness, nanoscale architectures, and sizes that are comparable to many biological molecules, they are of great interest in the biomedical area.^41^ Numerous applications in various areas of biomedicine have resulted from their intrinsic properties, which include electronic, optical, physicochemical, and surface plasmon resonance and can be changed by altering particle characteristics like size, shape, environment, aspect ratio, and ease of synthesis.^42^ These include photothermal and photodynamic therapy (PDT), imaging, sensing, targeted medication delivery, and the regulation of two or three applications.^43^ The current review highlights metal‐based NMs that have been reported for cancer applications. The purpose of this review is to explore the potential of NMs in cancer therapy so that new drugs can be developed for the diagnosis and treatment of cancer. We have also focused on plant‐based NMs that can be utilized in cancer treatment. This review article aims to inspire researchers to develop innovative approaches in the field of metal‐based NMs for cancer treatment and diagnosis. By highlighting the potential of these materials in various applications, such as drug delivery, imaging, and PTT, the article encourages researchers to explore and develop new strategies to enhance the potency and efficacy of metal‐based NMs. Ultimately, this could lead to the development of more effective and targeted cancer therapies, improving outcomes for patients.

## 2 APPLICATIONS OF METAL‐BASED NMS IN DIFFERENT TYPES OF CANCER

NMs hold a lot of promise to identify and eliminate cancer cells along with the potential to eradicate malignant cells with minor casualties to surrounding tissue.^44^ In terms of healthcare, there is growing optimism that NMs will lead to substantial advancements in illness treatment and detection. Researchers have been driven to create new nano‐platforms that can execute both activities at the same time as a result of these lofty ambitions. As a result, a new multidisciplinary study topic called theranostics has emerged. Iron‐based graphene oxide (GO), metal oxide magnetic nanocrystals, manganese dioxide (MnO_2_), black phosphorus, MXene, and palladium (Pd) are examples of theranostic NMs.^45^

NMs also assisted in the minimization of toxicity, optimization in targeting, maximizing the bioactivity, and offering a flexible way to modify the release of the encapsulated drug moiety.^46^ Meanwhile, inorganic NMs of different metals like Au, Ag, Fe, Ce, Se, Zn, and Ti are also used for their unique bioactivities in nanoforms. Tumor imaging plays an essential function in the detection and treatment of cancers like mammography. Potential applications of different NMs used in cancer therapy are given in Table 2.^47^

**TABLE 2:** Potential nanomaterials (NMs) used in cancer therapy.

Nanomaterial	Application	In vivo	In vitro	Physical property	Delivery	References
AuNPs	Tumor cell thermal ablation	No data	Supported	Photothermal energy conversion when aggregated	No data	^47^
AuNPs	Tumor cell thermal ablation	Supported	Supported	Radiofrequency‐generated heat	Local injection	^48^
AuNPs	Radiation therapy	Supported	Supported	High atomic number	EPR	^50^
AuNPs	Localized triggered drug release	No data	Supported	Size	No data	^51^
AuNPs	Dispersion of hydrophobic drugs in water	No data	Supported	Size	No data	^52^
AuNPs	Paclitaxel drug delivery	No data	Supported	Size, surface area	No data	^52^
AuNRs	Triggered release of multiple drugs	No data	No data	Photothermal energy conversion	No data	^54^
AuNRs	Localized triggered drug release	No data	Supported	Photothermal energy conversion	No data	^55^
AuNRs	Tumor cell thermal ablation	No data	Supported	Photothermal energy conversion	Active targeting (anti‐EGFR)	^56^
AuNSs	Tumor cell thermal ablation	No data	Supported	Photothermal energy conversion	Nano shell‐loaded monocytes	^57^
AuNSs	Tumor cell thermal ablation	Supported	Supported	Photothermal energy conversion	EPR‐active targeting (anti‐HER2)	^58^
AuNSs	Localized triggered drug release	No data	No data	Photothermal energy conversion	No data	^59^
Fullerenes	Photodynamic therapy	Supported	Supported	Formation of toxic radicals under irradiation	EPR	^59^
Hollow gold nanosphere	Tumor cell thermal ablation	Supported	Supported	Photothermal energy conversion	Injection active targeting (anti‐EGFR)	^60^
Liposome‐embedded magnetic NPs	Magnetically guided drug and gene delivery	Supported	Supported	Size, magnetism	Intravenous injection + magnetic guidance + active targeting	^62^
Liposome‐embedded magnetic NPs	Magnetically guided drug and gene delivery	Supported	No data	Size, magnetism	Intravenous injection + magnetic guidance	^62^
Magnetic NPs	Magnetically guided drug and gene delivery	Supported	No data	Magnetism	Magnetically guided	^64^
Magnetic NPs	Methotrexate drug delivery	No data	Supported	Size, surface area	Active targeting	^65^
Magnetic NPs	Tumor cell thermal ablation	Supported	No data	Neel relaxation	Local injection	^65^
Magnetic NPs	Localized triggered drug release	No data	No data	Neel relaxation	No data	^66^
Magnetic/Silica core/shell NPs	Dispersion of hydrophobic drugs in water	No data	Supported	No data	No data	^68^
Ni‐embedded carbon nanotubes	Magnetically guided drug and gene delivery	No data	Supported	Magnetism high surface area nanotube shape	Magnetic guidance	^68^
QDs	Photodynamic therapy	No data	Supported	Formation of toxic radicals under irradiation	No data	^70^
QDs	Drug delivery and monitoring of release	No data	Supported	FRET	Active targeting	^71^

Abbreviation: EPR, enhanced permeability and retention.

### 2.1 Transition metal nanosheets

Nanosheets are made up of different metals that have various applicabilities in different fields, such as chemotherapy,^71^ significant photothermal conversion efficiency, intensive near‐IR absorption, magnetic properties, and strong X‐ray attenuation properties.^72^ Different contrast agents, and transition metals, have been studied so far, because of their distinctive optical characteristics and low toxicity.^73^ The nanocomposites (NCs) are also reported to have an outstanding absorption in the near‐infrared region (NIR). Photoluminescence imaging has substantial implications for several transition metal NCs with small lateral dimensions.^74^ Transition metal NCs made up of large‐atomic‐number elements that had a high X‐ray absorption potential and might be used in computed tomography.

Transition metal dichalcogenides (TMDCs) have recently gained a lot of interest in nanomedicine because of their remarkable characteristics. A bottom‐up solution‐phase approach has been used to synthesize titanium disulfide (TiS_2_) nanosheets, a novel TMDC NM, which is modified using polyethylene glycol (PEG) and developed as TiS_2_‐PEG with good physiological stabilization and low in vitro toxicity.^76^

**FIGURE 1:**
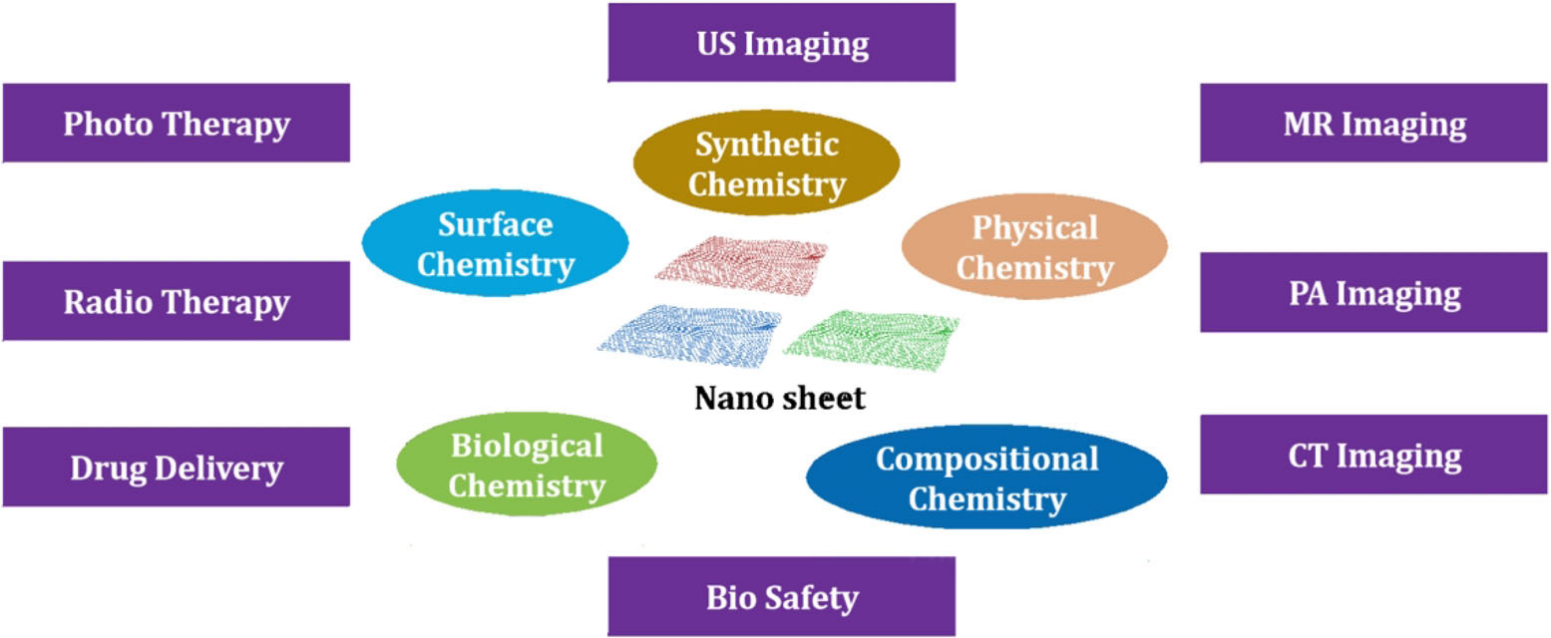
A pictorial diagram of the conventional 2D materials available for cancer diagnosis. 2D nanomaterials (NMs) with extraordinary physicochemical properties, large surface areas, and unique nanosheet structures have attracted tremendous interest in the field of nanomedicine. 2D NMs, such as black phosphorus nanosheets, metal–organic framework nanosheets, nitrides and carbonitrides, transition metal carbides, double‐layered hydroxides, and transition metal dichalcogenides, have been investigated in the area of nanomedicine.

The crystallinity of NMs is an important factor in determining their quality; however, measuring the crystal structures of thin TMDC nanosheets of 10 nm via standard in‐plane X‐ray diffraction (XRD) is difficult, and high‐resolution electron microscopy is usually required to directly determine these features. Due to their strong absorbance in the NIR, in photoacoustic imaging, TiS_2_‐PEG nanosheets could provide a significant contrast that showed the high tumor uptake. A pictorial diagram of the conventional 2D materials available for the cancer theranostics, and their oncological applications is given in Figure 1.^77^ In the field of cancer imaging, the use of NMs as contrast agents has garnered significant attention due to their unique properties. NMs can enhance the sensitivity and specificity of various imaging techniques, such as magnetic resonance imaging (MRI), CT, and PET. They can be engineered to specifically target cancer cells, thereby minimizing the risk of false‐positive results. QDs are an example of NMs that emit bright and stable fluorescence, making them ideal for optical imaging. Iron oxide NPs (IONPs), on the other hand, can be used as contrast agents for MRI and are biocompatible. AuNPs are highly biocompatible and can be functionalized with targeting molecules for both optical and CT imaging. Carbon nanotubes are highly sensitive contrast agents for both MRI and PET imaging. NMs have the potential to revolutionize cancer imaging by improving imaging techniques’ accuracy and specificity, leading to better diagnosis and treatment outcomes. They can also be utilized for targeted drug delivery, which can improve the efficacy of cancer treatment while minimizing side effects. Potential applications of different NMs used in cancer imaging are given in Table 3.

**TABLE 3:** Nanomaterials (NMs) used in the imaging of cancer.

Nanomaterial	Application	In vivo	In vitro	Physical property	Delivery	Reference
AuNPs	Early‐stage therapy monitoring	No data	Support	FRET	No data	^78^
AuNPs	Raman spectroscopy tags	Support	No data	Surface‐enhanced Raman scattering	Intravenous injection active targeting (anti‐EGFR)	^79^
AuNPs	Spectral‐domain optical coherence tomography	Support	No data	Strong scattering	Local injection active targeting (anti‐EGFR)	^80^
AuNPs	X‐ray contrast agents	Support	No data	Opaque to X‐rays	Intravenous injection	^81^
^192^Au colloids	Radio‐labeling	Support	No data	Radioactivity	No data	^82^
Gold nanocages	No data	Support	No data	SPR in NIR range	Intradermal injection	^83^
Magnetic/Au core/shell NPs	No data	Support	No data	Magnetism	Local injection	^84^
Magnetic NPs	MRI marker	Support	No data	Magnetism	Intravenous injection active targeting (anti‐HER2)	^85^
Nanoshell magnetic NPs	No data	No data	Support	Magnetism of magnetic NPs	Active targeting (anti‐HER2)	^86^
Paramagnetic NPs	No data	Support	No data	Magnetism	Active targeting	^87^
QDs	Lymph node tracers	Support	No data	NIR fluorescence	Intradermal injection	^88^
QDs	Cancer imaging	Support	No data	Photoluminescence	Intravenous injection active targeting	^89^

### 2.2 Magnesium oxide nanoparticles

Magnesium oxide (MgONPs) have unique properties that make them promising for cancer treatment. They are biocompatible and can induce programmed cell death (apoptosis) in cancer cells. MgONPs can inhibit the growth and proliferation of various types of cancer cells, including breast, colon, and lung cancer cells. They can enhance the efficacy of chemotherapy drugs by improving drug uptake and reducing side effects. MgONPs have also been used in PDT, producing reactive oxygen species (ROS) that can kill cancer cells when activated by light. However, further research is needed to determine their optimal dosage and delivery methods and ensure their safety in clinical settings.^90^ Several reports have been published so far regarding to MgONPs and associated microparticles for different activities like cellular uptake, oxidative stress, cellular apoptosis, genotoxicity, and cytotoxicity for various cancer and non‐cancer cell lines at different concentrations.^91^ According to the study, MgONPs were found to be excellent in the treatment of cancer cell lines and toxic only at high concentrations.^92^ MgONPs demonstrated significant binding interactions with human serum albumin (HSA) molecules via hydrophobic interactions and found to develop minor secondary structural alterations in the HSA molecules.^93^ MgONPs also reported for cytotoxicity against K562 cell lines and could be useful for delivering new anticancer drugs.^94^ MgONPs‐mediated apoptosis in cancer cells have been reported by the production of ROS.^91^ More research on MgONPs has been needed for anticancer activity. Rashad et al. synthesized MgONPs and ZnONPs by solid–solid reaction method and examined the structural properties of NPs by scanning electron microscopy (SEM) and XRD.^95^ The results have confirmed the synthesis of pure MgONPs and ZnONPs along with their size 70 and 50 nm, respectively. Ahmed et al. synthesized MgONPs by using a native bacterium *Enterobacter* sp. RTN2 and characterized by using microscopic and spectroscopic techniques.^96^ Karthikeyan et al. synthesized chitosan‐based MgONPs (CMgONPs) via a green precipitation process. The prepared CMgONPs were found spherical in shape, and the sizes of particles were 37 ± 2 nm. CMgONPs were investigated on MCF‐7 cell lines that showed excellent anticancer properties.^97^, ^98^ Behzadi et al. developed MgONPs that induced marginal changes in the secondary structure of HSA protein. The prepared MgONPs also exhibited potent cytotoxicity activity against K562 cell lines; due to this, these NPs could be counted as a novel agent for anticancer activity.^93^

### 2.3 Aluminum oxide nanoparticles

Aluminum oxides (Al_2_O_3_NPs) have been prepared by laser ablation, sputtering, sol–gel, pyrolysis, hydrothermal, and ball milling techniques. These NPs are generally round or nearly spherical in shape.^98^ Temiz and Kargın reported that Al_2_O_3_NPs interacted with tau proteins and created a static combination, which allowed these proteins to fold into more compact structures. Molecular docking and molecular dynamics simulation studies showed attachments of Al_2_O_3_NPs to the tau and facilitated some peripheral structural rearrangements.^99^

The cytogenetic effects of Al_2_O_3_NPs prepared from the plant *Allium cepa* were also evaluated for in vitro studies at a wide range of different concentrations. These studies demonstrated the increased numerous chromosomal abnormalities and a decrease in the mitotic index as a function of different Al_2_O_3_NPs concentrations.^100^ Al_2_O_3_NPs were also reported for the elevation in superoxide dismutases functioning corresponding to the different concentrations which counteract the ROS production. In another study, aluminum‐doped ZnONPs have been reported for anticancer activities against MDA‐MB‐231 cells via inducing cell death.^101^ However, Subramaniam et al. reported the anticancer effect of two different NPs, that is, zinc oxide (ZnONPs) and aluminum oxide (ANPs), in human colon carcinoma cells. These NPs (ZnONPs and ANPs) showed reduced cell proliferation. On the other hand, LDH leakage and colony formation were also assessed. They concluded that the ZnONPs and ANPs exhibited potent antiproliferative effects.^102^ Rajan et al. developed polyglutamic acid–modified AlNPs that were further fabricated and used as cytotoxic agents in human prostate cancer cells. AlNPs induced ROSs and mitochondrial dysfunction in PC‐3 prostate cancer cells that caused cell cytotoxicity.^103^

### 2.4 Palladium nanoparticles

Palladium (Pd) is a highly valuable metal that possesses catalytic, structural, and electroanalytical characteristics.^104^ Palladium (PdNMs) are being used as self‐therapeutics and have been shown to exhibit antibacterial and cytotoxic properties.^105^ When compared to other bacterial strains, *Escherichia coli*, PdNPs demonstrated a high level of growth inhibition against *Staphylococcus aureus*, indicating that they are useful against antibacterial agents, especially for gram‐positive bacteria.^106^ PdNPs mesoporous silica‐supported NMs were found to have moderately enhanced cytotoxic activity against human cancer cells.^107^ Against the ovarian cancer cells, PdNPs decrease the cell viability, increasing LDH leakage,^108^ enhancement of ROS generation, activation of autophagy, and cell death, with enhanced caspase‐3 activity followed by DNA fragmentation demonstrated the effectiveness of in vitro toxicity.^109^

ROS produced by PdNPs were the primary source of matrix metalloproteinase impairment, which increased the oxidative stress and cell death.^110^ Caspase‐dependent apoptosis is induced by PdNPs; the significant anticancer effect requires additional research into comparable PdNPs for the development of novel anticancer treatments. The in vivo and in vitro animal models of different types of cancer are needed to demonstrate the detailed mechanisms and therapeutic potential of PdNPs.^111^

### 2.5 Gold nanoparticles

AuNPs have been widely explored due to their remarkable biological and optical properties and can be employed for gene and drug delivery,^112^ as well as for the diagnostic, thermal ablation, and radiation augmentation purposes.^113^ Several chemicals are now being tested for possible medication delivery, especially in the treatment of cancer. The ability to change the surface of AuNPs with various targeting and functional chemicals significantly increases the variety of potential biological applications, with a focus on anticancer therapy. AuNPs have been reported to increase the solubility and retention time in blood, to help in the transportation of drugs, and control the release patterns of the therapeutics.^114^

Functionalized AuNPs have exceptional biocompatibility and predictable bioavailability characteristics that make them an excellent choice to deliver novel therapeutics.^52^ AuNPs used in PTT are controlled due to their unique shape, size, structure, and, most significantly, photothermal characteristics.^114^

**FIGURE 2:**
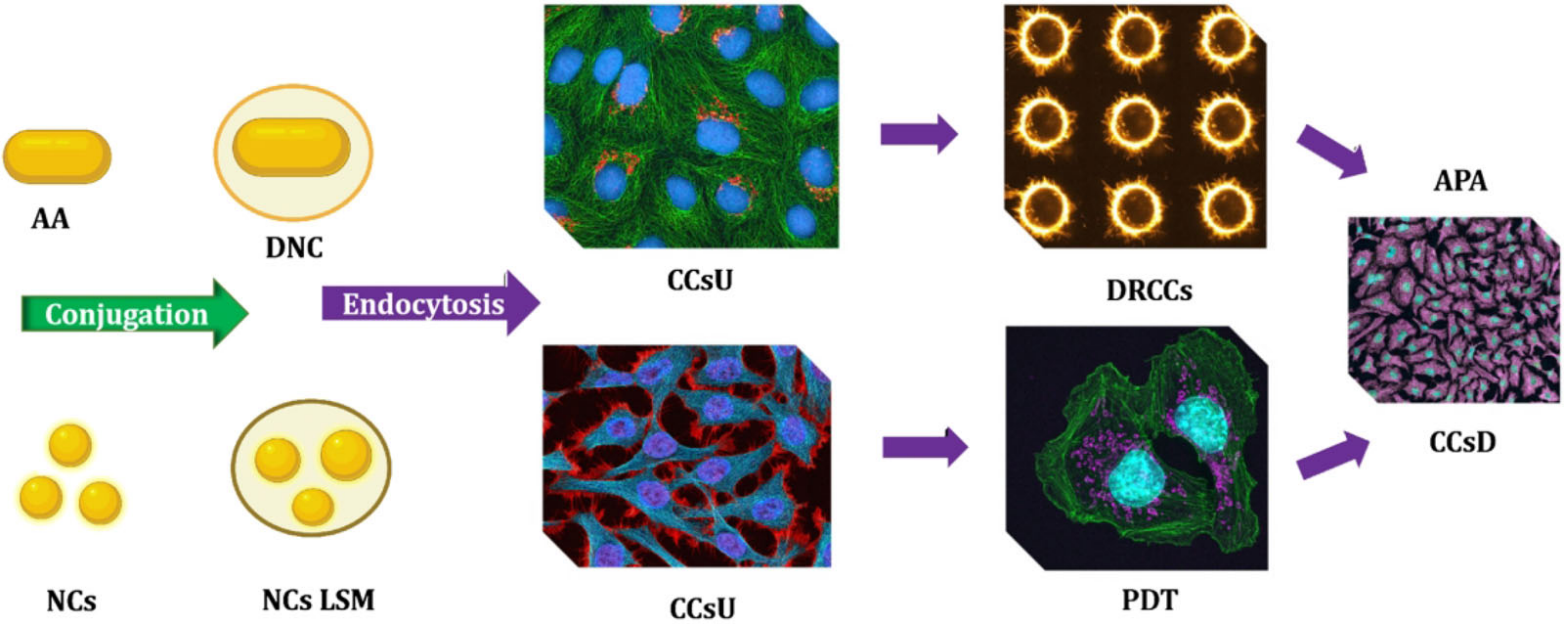
Mechanism involved in NCs based cancer therapeutics. AA, Anticancer agent; APA, Antiproliferative action of anticancer agent; CCsU, Cancer cells uptake; DNC, Drug loaded in nanocomposite; DRCCs, Drug release into the cancer cells; NCsLSM, Nanocomposites with light sensitive molecules; PDT, Photodynamic Therapy. [Source: National Cancer Institute, USA]

Gold nanorods (AuNRs) with different widths and lengths can affect the absorption and scattering band from visible to NIR. Thickness and core radius of AuNPs can produce a comparable result.^116^ This allows the development of AuNRs and gold nanoshells (AuNSs) with unique properties according to the tumor's size and location.^117^ Furthermore, the aggregations of AuNPs have been found to have a considerable influence on both optical and thermal characteristics.^118^ A pictorial diagram with the concept of AuNPs in the cancer treatments is provided in Figure 2.^119^

Multifunctional nano platforms that integrate therapeutic elements and multidisciplinary imaging are the future of nanomedicine.^120^ The ultimate goal for NP‐based medicines is to enable efficient, specific in vivo drug delivery without systemic toxicity, with the dose given, and therapeutic efficacy over time.^121^

**FIGURE 3:**
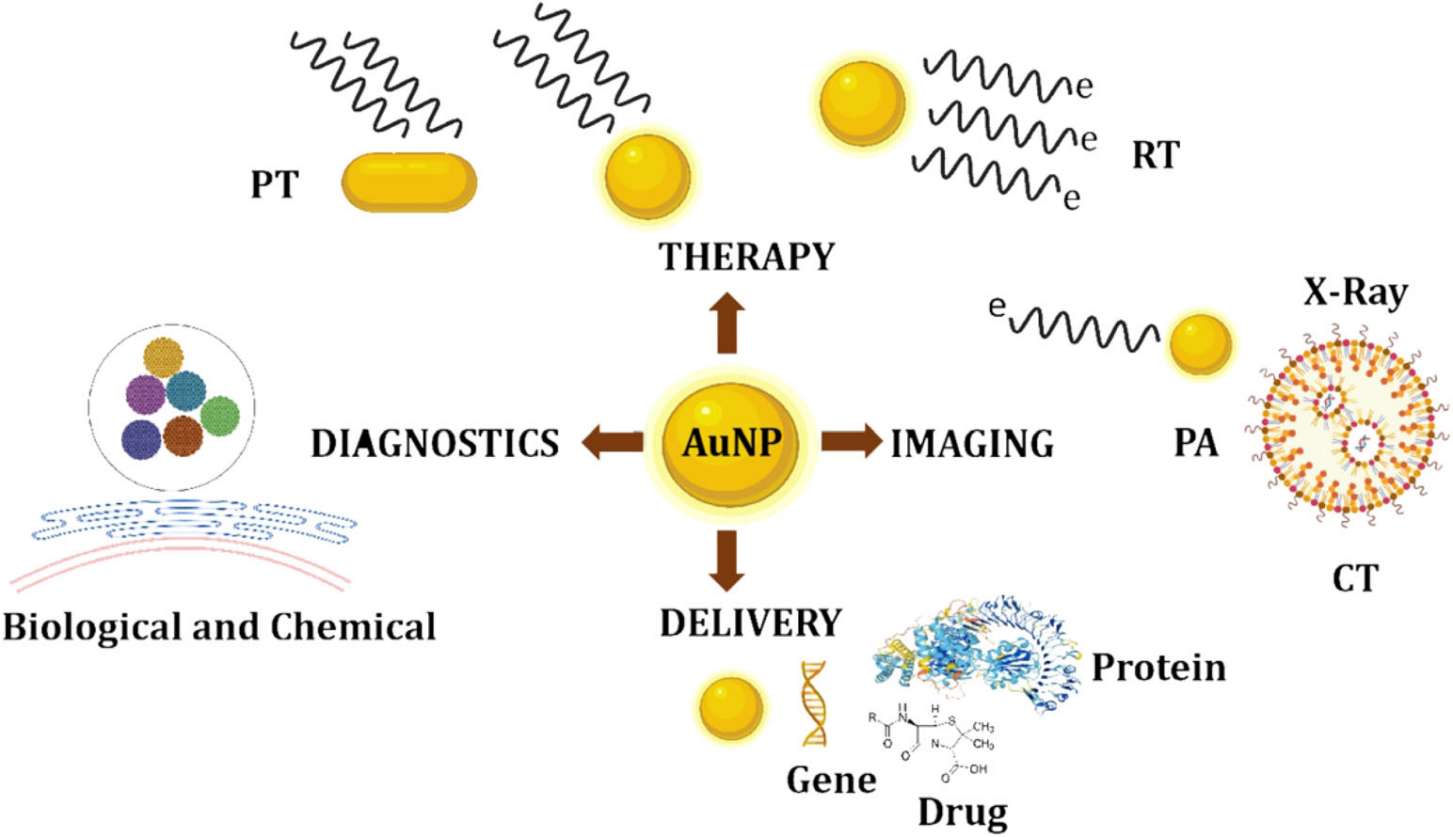
AuNPs have biomedical significance due to their electronic, physicochemical, and unique optical properties. The methods that enable for real‐time and high‐quality imaging in in‐vivo optical imaging are used to study biodistribution in living animals. Nuclear medicine Imaging, CT, and magnetic resonance imaging (MRI). Quantative data of uptake into specific organs or tissues can also be obtained by using PET and SPECT. The approaches provided include LSC and indirectly assessing drug concentration, only in in‐vivo optimum imaging, such as CT and MRI, can image nanoparticles (NPs) biodistribution in longitudinal studies at several points.

Functionalized AuNPs with therapeutic and targeted peptides could be a promising anticancer nanosystem for improving therapeutic drug efficacy.^122^ To avoid filtration in the spleen, the size of AuNPs must be less than 150 nm. The particles should be larger than 15 nm to avoid fast clearance by the kidneys; hence, 90 nm is in the proper size range. For AuNPs, the best size is reported around 90 nm for cell targeting.^123^ DDSs of this sort can be designed to carry one or more medications as well as many targeting compounds as therapeutic and diagnostic NPs for imaging and therapeutic purposes.^124^ AuNPs have been used in several applications, such as delivery, therapy, imaging, and diagnostics application given in Figure 3.

### 2.6 Copper oxide nanoparticles

Copper oxide (CuONPs), a brownish–black powder, exposed to hydrogen or carbon monoxide at elevated temperatures, can be converted to metallic copper with controllable size and desirable characteristics.^125^ CuONPs can be modified to generate innovative and desired results. However, chemical and electrochemical methods of NPs synthesis are generally more efficient. On the other hand, biogenic methods for CuONPs synthesis are gaining popularity due to their lower cytotoxicity.^126^ The use of CuONPs for the identification of biomarkers for cancer, diabetes, hypoxia, stress, cardiac syndromes, and neurological complications was investigated.^127^

CuONPs open up a new scope for the establishment of in vivo and in vitro sensing and therapeutic applications.^128^ CuONPs demonstrated the potency could be boosted in a composite form to merge the various functionalities. CuONPs have lately been examined in various in vivo and culture cell lines due to their cytotoxic capabilities and gained a lot of focus in cancer treatment.^129^

Fahmy et al. synthesized CuONPs gilded with GO by using a green synthesis method, and cytotoxic activity was found to be excellent against HCT‐116 cell lines at 100 μg/mL.^130^ Kouhkan et al. synthesized CuONPs from *Lactobacillus casei*., and results confirmed the anticancer effects of these NPs that were investigated by the Griess test and methylthiazolyldiphenyl‐tetrazolium bromide (MTT) assays.^131^ This study demonstrated that the cell viability of human cancer cells decreases when treated with CuONPs and suggested that CuONPs may be a potential anticancer agents.^132^

### 2.7 Nickel nanoparticles

Nickel (NiNPs) have been used as catalysts, high‐density magnetic recording media, as well as other applications due to their remarkable structure and functionality.^133^ Chen et al. investigated how NiNPs and the anticancer medication verbascoside (VB) worked together to induce apoptosis in K562 cells, and by using an electrochemical experiment showed that NiNPs significantly aided the uptake of VB into K562 cells.^134^ Exposure with VB‐Ni significantly activates apoptosis in K562 cells, as evidenced by apoptosis labeling and DNA fragmentation. The administration of VB‐NiNPs substantially prevented the growth of tumors in mice, according to in vivo experiments.^135^ Increased cell apoptosis was found to be directly linked to improved tumor growth inhibition in the animal study. VB‐NiNPs could be a revolutionary technique for carefully monitoring cancer cells in order to effective cancer treatment.^136^

Metallic NiNPs were shown to be more cytotoxic than fine particles in an investigation, and small particles were found to activate phospho‐Akt and Bcl‐2 more than coarse particles. Metallic NiNPs have different physicochemical characteristics than the bulk material of the same concentration. Unfortunately, very less data is available about the pathways involved in the carcinogenic consequences of metallic NiNPs.^137^

NiNPs were also reported in a mouse model of malignant breast cancer, which demonstrated that the NiNPs are responsible for the suppression of tumor cell growth in the mouse model.^133^ Further suggested that NiNPs may be helpful in the discovery and development of new anticancer nanomedicine.^132^ NiNPs and their shape were found to be spherical along with the size of 16.85–49.04 nm by incorporating the aqueous extract of *Fumaria officinalis*. The cytotoxicity of NiNPs and antihuman ovarian cancer activity was evaluated on the SW‐626, Caov‐3, SK‐OV‐3, and PA‐1 cell lines.^137^ The study further suggested that NiNPs along with *F. officinalis* leaf aqueous extract may be useful in the formulation of novel anticancer drugs for the diagnosis of cancer.^138^

### 2.8 Zinc oxide nanoparticles

Zinc oxide (ZnONPs) have a lot of surface area due to their size and catalytic activity.^139^ The physical and chemical properties of ZnONPs are based on how they are manufactured. It has been reported that ZnONPs have potential inhibitory effects on cancer cells due to their inherent toxicity.^140^ It can be obtained via resulting intracellular ROS formation and stimulating the apoptotic signaling mechanism. ZnONPs have been demonstrated to enhance the bioavailability of nanomedicine and resulted in the improvement of chemotherapy.^141^

The uses of ZnONPs as DDSs for loading and delivering hydrophobic anticancer agents are being investigated, and combining it with PBA improves its absorption in tumor tissue via interacting with the sialic acid.^140^ In HepG2 cells, ZnONPs also caused caspase‐3 activity, DNA breakage, ROS production, oxidative stress, and according to research ZnONPs specifically triggered apoptosis in cancer cells.^142^ The cytotoxicity of ZnONPs against several types of cancer cells has been studied.

According to the findings, ZnONPs have unique effects on mammalian cell viability and killed different types of cancer cells while not affecting normal rat astrocytes and hepatocytes.^143^ It has been reported that ZnONPs demonstrated the cancer cell–specific toxicity via the destruction of mitochondrial membrane potential and formation of ROS, which results in the stimulation of caspase cascades, and apoptosis of cancer cells.^144^ ZnONPs are used as an important carrier for the sustained delivery of several plant‐based chemotherapeutics and bioactive compounds for targeting tumor cells.^145^

### 2.9 Titanium dioxide nanoparticles

Titanium dioxide (TiO_2_NPs) were first prepared in the 1990s and have been used in a variety of biomedical disciplines, such as tissue engineering and the production of medicinal drugs.^146^ TiO_2_‐based DDSs have proved their capacity to reduce tumorigenesis risk and improve cancer therapy in recent years. Titanium (TiNMs) mainly are including titanium oxides, titanium sulfides, titanium hydrides, titanium nitrides, titanium carbides, and titanium‐organic compounds.^147^ Complexes of TiO_2_NPs offer a new perspective on chemotherapy; multiomics methods based on TiO_2_NPs have universally matured to target structural, molecular, and phenotypic concentrations.^148^

TiO_2_NPs have been reported for several types of cancer treatment and are used in medical applications and anticancer drug development. Their uses are found to be in the SDT, PDT, and DDSs.^149^ It has been reported that the expression of caspase‐9 and caspase‐3 was significantly found to be increased at several concentrations of TiO_2_NPs for 48 h. Further studies demonstrated that TiO_2_NPs were found to be inhibiting A549 cell proliferation and responsible for DNA damage along with the introduction of apoptosis.^150^

Wang et al. provided the assay and scientific evidence that TiO_2_NPs can damage DNA and induce significant cytotoxicity and apoptosis of A549 cells. TiO_2_ results in the formation of an array of ROS.^151^ This mechanism is responsible for cell death and found several applications in the PDT for the diagnosis of cancer. It has been reported that TiO_2_NPs were examined as photosensitizing agents in the diagnosis of malignant cells. TiO_2_NPs along with their NCs and combinations with another bioactive molecule can be used as photosensitizers in PDT.^152^

### 2.10 Silver oxide and silver nanoparticles

Silver oxide (Ag_2_ONPs) were prepared by using chemical and biological approaches.^153^ Ag_2_ONPs are a remarkable material with a lot of potential in biomedical applications. An aqueous dispersion of Ag_2_ONPs produced by treating water with flashed electrical discharges effectively prevented tumor growth in in vivo.^154^ The prolonged systemic influence of Ag_2_ONPs resulted in tumor growth regression in some cases, as demonstrated by histomorphological research.^155^

The phototoxic and cytotoxic effects of Ag_2_ONPs were investigated using a variety of relevant experimental approaches on a hepatocellular (HepG2 cell line) model. The acquired results were validated by the use of polynomial fit, which confirmed the quality of fit.^156^ As an anticancer agent, Ag_2_ONPs have unique bio interaction features and physicochemical properties, and because of their confined drug qualities at the needed region, Ag_2_ONPs are considered to be a potential anticancer agent.^157^ Karunagaran et al. synthesized spherical shaped along with the size of 30 nm Ag_2_ONPs by using *Bacillus thuringiensis* culture supernatant and evaluated their cytotoxic effect by MTT assay against Chang liver and HepG2 cell lines.^158^ Ag_2_ONPs demonstrated the dose‐dependent response on both of the cell lines. The study further suggested that Ag_2_ONPs may have excellent anticancer potential. Abbasi Kajani et al. prepared Ag_2_ONPs from ethanolic and aqueous leaves extract of *Rhamnus virgata* in a facile, green, cost‐effective way, and their anticancer potential was evaluated by using HUH‐7 and HepG2 cell lines.^159^ Significant anticancer properties were found from the study, which was further suggested that Ag_2_ONPs may be useful in the development of potential anticancer nanomedicines.^160^

### 2.11 Iron oxide nanoparticles

IONPs are tiny particles made of iron oxide, which is a compound of iron and oxygen.^161^ They have a wide range of potential applications, including in medical imaging, magnetic hyperthermia for cancer treatment, and as catalysts in chemical reactions.^162^ Due to their small size, IONPs have unique properties, such as large reactivity, high surface area, and strong magnetic properties. However, their potential toxicity also needs to be considered when used in biological systems.^163^ IONPs have been also studied for their potential use in cancer imaging, specifically, MRI.^164^ MRI is a powerful imaging technique that can provide detailed images of internal organs and tissues, including tumors. However, the contrast in the images can be improved by using IONPs as contrast agents.^165^ IONPs can be functionalized with targeting agents, such as antibodies or peptides, which specifically bind to cancer cells. These functionalized NPs can then be administered to the patient and will accumulate in the cancerous areas, providing a strong contrast enhancement in the MRI images.^166^ This can make it easier to detect and monitor the progression of cancer. Additionally, IONPs can also be used for in vivo tracking of cells and therapeutic agents, for example, stem cells. It is worth noting that although IONPs have great potential in cancer imaging, more research is needed to fully understand their safety and efficacy before they can be widely used in the clinic.^167^ Moreover, there are other imaging modalities, such as PET–MRI that can also be used to detect cancer; IONPs can be used as tracer in these modalities as well.^168^ IONPs have been studied for their potential use in cancer treatment.^169^ One of the most promising applications is magnetic hyperthermia, which involves heating cancer cells using an alternating magnetic field to generate heat within the NPs.^170^ This heat can damage or kill the cancer cells while minimizing harm to healthy tissue. Additionally, IONPs can be functionalized with targeting molecules, such as antibodies or peptides, to specifically deliver drugs or other therapeutic agents to cancer cells; this is known as magnetic targeted therapy.^171^ This can help to increase the effectiveness of treatment while reducing side effects. Moreover, IONPs can also be used in imaging, such as MRI, to help identify and monitor the progression of cancer. IONPs can be functionalized with targeting agents to specifically target cancer cells, enhancing the contrast in the image, and making it easier to detect and monitor the cancer.^172^ IONPs have been studied for their potential use in cancer therapy. IONPs can be targeted to cancer cells using antibodies or other targeting molecules, and they can also be directed to tumors using magnetic fields.^173^ Once they reach the cancer cells, IONPs can be used to deliver drugs or heat to the cancer cells, which can help to destroy them. IONPs have also been studied as a way to enhance the effectiveness of radiation therapy by increasing the amount of radiation that reaches the cancer cells.^174^ However, more research is required to understand the therapeutic potential of IONPs in the chemotherapy, and more clinical studies are needed to investigate their efficacy and safety.^175^ Kanipandian and Thirumurugan synthesized AgNPs from the leaf extract of *Gossypium hirsutum* to treat A549 lung cancer cell lines. The NPs were formulated using the extract in a 1 mM solution of silver nitrate. These AgNPs were found with a spherical shape having the size range of approximately 40 nm. Staining with dye HOECHST 33342, the effect of AgNPs was observed on cell lines for 3 days every 24 h. The enhanced apoptosis was observed in AgNP‐treated cell lines as compared to normal cells, which was confirmed via direct fluorescence microscopic analysis. Meanwhile, AgNPs were also observed for changing the proteins’ properties responsible for processes such as pro‐ and antiapoptotic for a time‐dependent manner where the levels of p53 expression have been increased.^176^

## 3 SILICON DIOXIDE NANOPARTICLES

Silicon dioxide (SiO_2_NPs) are commonly utilized in biomedicines for different diseases such as cancer as well as consumer items, including cosmetics and sunscreens.^177^ SiO_2_NPs may be useful for the development of potential nanomedicine due to their unique structure, a size that incorporates several functions to utilize optical properties. SiO_2_NPs can be used in optical imaging due to their different optical characteristics at the micro‐, nano‐, and in vivo levels using both visible and NIR.^134^ However, depending on the size and concentration of NPs, SiO_2_NPs can have some harmful impacts on human health.

SiO_2_NPs with a thyroid‐stimulating hormone receptor‐targeting ligand can effectively target thyroid malignancy.^178^ In vivo, SiO_2_NPs reduce tumor size while having fewer harmful side effects. This study lays the door for efficient thyroid cancer chemotherapy.^179^ The cytotoxic effect of SiO_2_NPs was evaluated on MCF‐7 cell lines by increasing the concentration of NPs. The apoptosis rate was found to be increased, and the proliferation rate was reduced.^180^ In this study, antitumor effects of SiO_2_NPs were found to be excellent and presented as good gene carriers for breast cancer. SiO_2_NPs were also reported for the improved release rate of gene drugs and enhanced encapsulation efficiency. It has been observed that as increasing the concentration of SiO_2_NPs, its effect on breast cancer cells was found to be increased, whereas the damage on the normal cell was minimum.^181^ This study suggested that SiO_2_NPs may be an excellent targeted gene DDSs. Asiri et al. synthesized SiO_2_NPs conjugated with (3‐glycidyloxypropyl)trimethoxysilane (3GPS), and these NPs were characterized on the basis of size, morphology, SEM, thermogravimetric analysis, Fourier transform infrared spectroscopy (FTIR), and transmission electron microscopy (TEM).^182^ In this study, it was found that SiO_2_NPs possess potential anticancer properties against colon cancer.^183^

SiO_2_NPs were also investigated on HCT‐116 to evaluate their anticancer potential using an MTT assay. Results demonstrated that cancer cell viability and cell proliferation were found to be decreased, and SiO_2_NPs caused cell death in a dose‐dependent way. SiO_2_NPs provide application in the therapy of certain diseases, in vivo imaging, and cancer diagnosis.^184^ Further suggested that SiO_2_NPs may have applications in photodynamic therapy, in which a lesion location is treated by irradiating it with light. The potential for SiO_2_NPs to play an essential role in the integration of light‐based diagnostics and treatments.^177^

## 4 SELENIUM NANOPARTICLES

Selenium (Se) is a trace mineral used to develop selenium (SeNPs) that have gained importance in the research due to their potential anticancer properties.^185^ Human dermal fibroblasts cells at quantities up to 1 ppm, the SeNPs demonstrate mild cytotoxicity while having anticancer activity on human melanoma as well as glioblastoma cells at the same range of concentrations.^186^ Several selenoproteins contain oxidoreductase function and, hence, control the redox balance in the body. Se has limited treatment efficacy and narrow toxicity margins, but SeNPs have significantly reduced toxicity.^187^

SeNPs have been studied for possible therapeutic benefits in a variety of oxidative stress. To the inflammation‐mediated diseases, such as diabetes, cancer, nephropathy, and arthritis, and provide an acceptable transport platform for transporting various drugs to the point of activity,^188^ SeNP‐based techniques have shown promise in combating drug resistance and minimizing the side effects of chemotherapeutic drugs. SeNPs provide a great platform for transporting chemotherapeutic agents to the targeted area. In comparison to MDA‐MB 231 cells, it also influences estrogen receptor signaling in MCF‐7 cell lines that result in the enhanced production of cytochrome C, Bax, and P‐p38.^189^ In MCF‐7 cells, SeNPs were found to have lower binding and promote apoptosis, necrosis, and decrease CD44 expression; they also caused disruption and deregulation of the cytoskeleton F‐actin.^190^ In fibrosarcoma cell lines, SeNPs decrease the production of matrix metalloprotein‐2, which is associated with tumor invasion, metastasis, and angiogenesis. Xia et al. developed doxorubicin‐based SeNPs for the treatment of non‐small lung cancer cell lines.^191^ Modification of SeNPs was performed via the addition of cyclic peptide, Arg‐Gly‐Asp‐d‐Phe‐Cys [RGDfC]. After the modification, doxorubicin drug was loaded, and the RGDfC‐Se@DOX system was prepared for targeting cancer cell lines. Different characterizations such as TEM and FTIR were performed, and the sizes of the particles were found to be 18 nm, having 7–12 nm average spherical particle size. Flow cytometry was performed for the investigation of apoptosis induced in the A549 cell lines. The resulted RGDfC‐Se@DOX system showed higher apoptosis as compared to the DOX and Se@DOX groups. This study concluded that the nano delivery system played a crucial role in the treatment of lung cancer.^192^, ^193^

## 5 NANOCOMPOSITES WITH MULTIPLE APPLICATIONS

Nanocomposite (NC) materials are made up of many phases, each of which has at least one, two, or three nanometer‐scale dimensions.^194^ Understanding the structure–property relationship is directly influenced by the ratio of surface area to volume of reinforced material utilized during NCs synthesis.^195^ NCs provide us with numerous ideas for addressing problems in a variety of fields, including healthcare, pharmaceuticals, food packaging, electronics, and energy.^196^ The physical characteristics of the NCs could be useful to develop new and effective cancer‐detecting sensors, tumor‐imaging agents, and cancer treatment medicines.^197^

Biocompatible polymers with inorganic NPs and natural or rationally designed biomolecules provide a path toward multifunctional composite systems.^198^ Although just a few of these innovations have been tested in humans, NCs based on functionalized semiconductors and metal have the potential to improve the cancer diagnosis and treatment.^199^ With the right composition, size, and 3D structure, NCs could be used as a powerful immune adjuvant system.

Zinc oxide NCs (ZnONCs) are rapidly taken up by antigen‐presenting cells due to their excellent physical and chemical properties and decomposed to release intracellular Zn ions and, as a result, cause the formation of intracellular ROS.^200^ Toll‐like receptors identify NCs, which effectively activate innate immune cells and drive pro‐inflammatory responses. These NCs improve antigen‐specific adaptive immune responses, such as antibody formation and T cell responses,^201^ by activating antigen‐presenting cells and delivering associated antigens into intracellular compartments. Various NPs capable of targeting the cell nucleus are given in Table 4.^202^

**TABLE 4:** Various nanoparticles capable of targeting the cell nucleus.

Nanoparticles	Targeting mechanism	Size (nm)	Reference
Au nanorods	NLS: TAT peptide	10 × 40	^203^
AuNPs	NLS: SV40 large T antigen	29 ± 3	^204^
AuNPs	Positive charge	13	^205^
AuNPs	Passive diffusion	8.5	^206^
AuNPs	Passive diffusion	2, 6	^206^
Chitosan NPs	Nuclear receptor: dexamethasone	25, 50	^208^
Compound NPs composed of the PEG–benzoic	NLS: oligo‐l‐lysine	150	^209^
Copolymer NPs—poly(oligoethylene glycol methacrylate)‐block‐poly(styrene‐co‐vinylbenzaldehyde)	Passive diffusion	Rods: 5–10 × 100–300 Worms: 5–10 × 400–700	^210^
Hydroxyapatite NPs	Passive diffusion due to cell apoptosis	78	^211^
imine–oligo‐l‐lysine/iridium(III) metallodrug complex	NLS: CPKKKRKV	40	^212^
Iron oxide nanoparticle core/mesoporous silica shell	NLS: TAT peptide	50	^213^
Mesoporous organo silica NPs	NLS: TAT peptide	30	^214^
Mesoporous silica NPs	NLS: TAT peptide	25	^215^
Mesoporous silica NPs	NLS: TAT peptide	40	^216^
Prussian blue NPs and AuNRs	NPs open the nuclear membrane	50 and 10 × 100	^217^
Upconversion nanoparticle core/silica shell	NLS: TAT peptide	50	^218^
Upconversion nanoparticle core/hollow mesoporous silica shell	NLS: TAT peptide	50	^219^
Upconversion nanoparticle core/TiO2 shell	NLS: TAT peptide	31	^220^

In vivo research using ZnONCs with distinct compositions produced encouraging proof of concept data for developing therapeutic vaccinations against infections and malignancies.^221^ The use of NCs as vaccine delivery systems and immunotherapeutic agents is still in the early stages of research. Several difficulties are encountered, including challenges in mass‐producing NCs with consistent functionalities and acceptable capabilities in a reproducible manner.^222^ Due to a lack of expertise in nano–bio interfacial interactions, further research is needed to determine the possible toxicity and biodistribution of NCs during in vivo usage.

## 6 PLANT‐BASED NANOMETAL IN CANCER

The benefits of employing plants to synthesize NPs are they remain effectively accessible, safe, and produce a variety of metabolites that may be useful in cancer treatment.^222^ A variety of plants are presently being studied because of their significance in NPs synthesis.^223^ AuNPs were synthesized by using an *alfalfa* plant with a size range of 2–20 nm.^224^ Ag, Ni, Co, Zn, and Cu NPs also have been synthesized inside live *Brassica juncea* plants (Indian mustard), *Helianthus annuus* (sunflower), and *Medicago sativa* (Alfalfa).^225^ Several plants are known to aggregate metal concentrations that are higher in concentrations, in comparison to others; these are referred to as hyper‐accumulators of the plants studied. The *B. juncea* exhibited a superior potential to accumulate metals and eventually assimilate them as NPs.^226^ A lot of work has been done recently in terms of plant‐based reduction of metal NPs, as well as the significance of phytochemicals. The primary important phytochemicals have also been discovered as carboxylic acids, amides, aldehydes, ketones, flavones, and terpenoids in the wavelength of infrared spectroscopic studies.^227^ Flavones, quinones, and organic acids are the main water‐soluble phytochemicals that contribute to immediate reduction.^228^ The phytochemicals present in *Hydrilla* sp. (hydrophytes), *Bryophyllum* sp. (xerophytes), and *Cyprus* sp. (mesophytes) and their significance in the synthesis of AgNPs are investigated.^229^ The Xerophytes were confirmed to have emodin, an anthraquinone that undergoes the tautomerization that leads to the productions of AgNPs.^230^ Catechol is reported to be converted into protocatechualdehyde and protocatechuic acid under alkaline environments. Reactions resulted in the liberation of hydrogen, and it was concluded that it was involved in the production of NPs.^231^ The NPs synthesized using hydrophytes, mesophytes, and xerophytes were found to be in the size of 2–5 nm. Some of the plant‐based NPs are listed in Table 5.^232^

**TABLE 5:** Plant‐based nanometals in the cancer diagnosis.

Plant name	Nanometal/NPs	NPs size (nm)	NPs shape	References
*Alfalfa plant* (*Medicago sativa*)	Au and Ag	20–40	Triangular and spherical	^234^
*Aloe vera*	Ag	15–15.6	Spherical	^235^
*Avena sativa*	Au	25–85	Spherical	^236^
*Azadirachta indica*	Ag, Au	50–100	Spherical	^237^
*Capsicum annum*	Ag	16–40	Spherical	^238^
*Cinnamomum camphora*	Au and Ag	55–80	Spherical	^239^
*Emblica officinalis*	Au and Ag	10–20 and 15–25	Spherical	^240^
*M. sativa*	Zn	2–5.6	Spherical	^241^
*M. sativa*	Ni/Ti	2–6	Spherical	^242^
*Tamarind leaf extract*	Au	20–40	Spherical	^243^

Abbreviation: NPs, nanoparticles.

## 7 THE EFFECT OF SIZE, SHAPE, AND SURFACE PROPERTIES OF NANOPARTICLES

**FIGURE 4:**
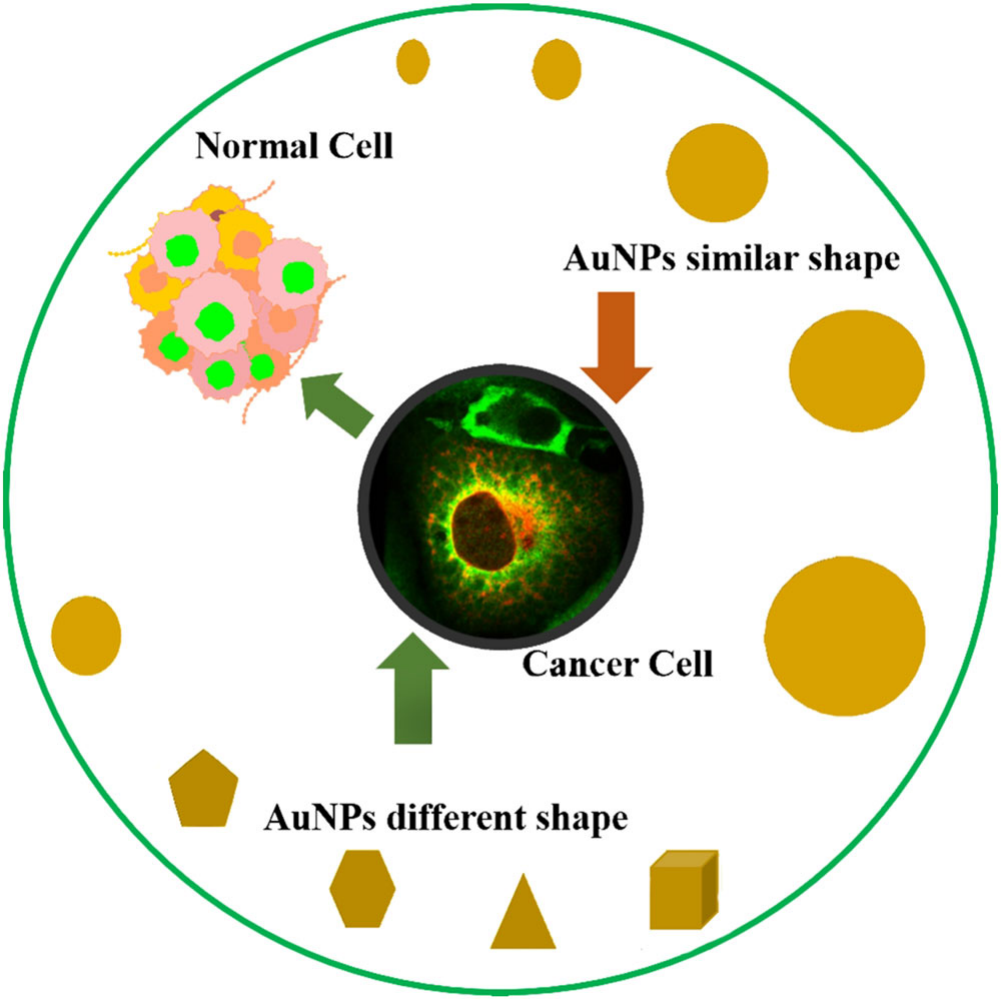
AuNPs having similar shapes, different sizes, and different shapes, same sizes targeting cancer cells. Gold nanoparticles (AuNPs) have been found to effectively target cancer cells of varying shapes and sizes, and their targeting abilities and biological effects can be tailored for different cancer applications. For example, spherical AuNPs of varying sizes can affect their effectiveness as drug delivery agents due to different cell uptake and distribution patterns, whereas differently shaped AuNPs can target different cancer cell types, potentially improving the specificity and efficacy of cancer treatment. [Source: National Cancer Institute, USA]

Vascular permeability diminishes as the size of NPs increases.^244^ The tumor vesicles have different hole sizes depending on the type of tumor and where it is growing. For example, the pore diameters of malignancies in the cranial window are significantly smaller than those found in the dorsal chamber; the size of the NPs must be adjusted to the type and location of the tumor.^245^ The shape, size, and core chemical characteristics of NPs are all important factors in cellular uptakes, like spherical, cubic, rod shape, or worm shape, which have a significant impact on cellular uptake and anticancer properties.^246^ When cubic, spherical, and rodlike AuNPs are compared, spherical NPs found to have the higher uptake in terms of weight, and rod‐shaped NPs demonstrate the large uptake in terms of quantity.^247^ The circulation period improved as the size of AuNPs increased from 5 to 25 nm. NPs aggregate into solid tumors in cancer treatment by utilizing the enhanced permeability and retention (EPR) effect.^248^ However, the circulation time of NPs has a major impact on EPR. Large NPs have significantly longer circulation durations, which enables them to take advantage of the EPR effect. Therefore, for NPs that rely on the EPR effect to localize to tumors, particle size is important. A pictorial diagram of different shapes of AuNPs along with similar sizes and similar shapes along with different sizes targeting cancer cells is given in Figure 4.^249^

The 50 nm size of AuNPs also affects cellular uptake due to their influence on the entropic and enthalpic characteristics, which control the efficiency of AuNPs adherence to cellular receptors.^250^ It has been found that AuNPs with a size of 50 nm demonstrated the highest cellular uptake, which is required for chemotherapies.^251^ Spherical and smaller NPs resulted in deeper, more uniform penetration inside the solid tumor, and the shape and size ratio of NPs may be important parameters in the development of better penetrating, more efficient, nano‐carriers for cancer therapies.^252^ The anticancer efficiency of AuNPs was found to be in increasing order: stars, rods, and triangles. The mechanisms of cellular uptake of AuNPs were investigated and found that several sizes and shapes of AuNPs tended to use different endocytosis pathways.^253^ Triangle AuNPs were found to be more effective than other NPs; the size limit of 30 nm for spherical AuNPs indicates that NPs smaller than 30 nm are not effective to drive the membrane process. AuNPs in the size range of 2–100 nm decorated with Herceptin were found to be effective in breast cancer.^254^

Due to the size limitation of the NPs, a size of 60 nm in diameter results in a shortage of receptors, and size below 10 nm is found to be rapidly eliminated.^255^ Several studies reported that NPs with smaller size and rod shapes have higher binding potential due to larger contact areas and smaller drag forces.^256^ In the in vivo environment, AuNPs with different sizes and shapes interact in a different way, and several studies demonstrated that spherical AuNPs can improve interaction with the immune system and stimulate toxicity, due to the similarity in shape and size of pathogenic microorganisms. When considering the entry of AuNPs in the cells, both shape and size will be very essential.^257^

Cell receptors are found to be in different shapes and sizes; due to this, there will be an advantage to design AuNPs that can perfectly fit into the receptor site.^257^ A size of 50 nm demonstrates the higher cellular uptake, whereas a size of 20 nm or less shows the excellent tumor penetration.^258^ NPs in the size of 23 nm improved in the cellular uptake than the size of 85 nm for silica dye–doped NPs.^259^

Using NPs in the medical sciences would result in improved therapeutic efficacy, easier drug administration, fewer unwanted side effects, lower systemic doses, and enhanced patient quality of life and compliance.^261^ To explore the NPs in clinical research, further research is required to help in the understanding of the interaction between the NPs and biological systems.^262^

The surface properties of NPs are critical for their behavior in in vivo and must be carefully considered in the design and development of NPs for in vivo applications.^263^ The surface of NPs can have a significant effect on their behavior in the in vivo. Their unique surface properties, such as the surface charge, functional groups, and chemical composition, can affect the interactions of NPs with cells and biomolecules in the biological system.^264^ The most important aspects of the surface of NPs are the presence of coating or functional groups. These can play a critical role in the targeting and uptake of NPs by cells and can also affect the circulation time and stability of NPs in the biological system.^265^ For example, the presence of specific targeting molecules such as peptides or antibodies on the surface of NPs can improve their selectivity for specific tissues or cells. Another important aspect of the surface of NPs is the chemical composition, which can affect the toxicity and biocompatibility of the NPs.^266^ For example, NPs made of biodegradable polymers such as PEG can have a reduced toxicity profile and a longer circulation time in the biological system.^267^ The surface charge of NPs can also affect their behavior in the in vivo. The presence of negatively charged NPs tends to have a higher degree of stability in biological fluids and can be taken up more efficiently by cells, whereas positively charged NPs can have a greater tendency to aggregate and be rapidly cleared by the reticuloendothelial system (RES).^268^ NPs can exhibit a variety of behaviors in vivo, depending on their size, shape, composition, and surface properties. Several NPs are taken up by cells and can be used for targeted drug delivery, whereas others are rapidly cleared from the body by the immune system.^269^ The behavior of NPs can also be influenced by their interactions with biomolecules such as proteins and lipids in the body. The NPs can have different biodistribution patterns, depending on the route of administration, such as oral, topical, or intravenous injection. Some NPs can have specific targets such as cancer cells, and they can also have different ADME and pharmacodynamics. The charge of NPs can have a significant effect on their behavior in vivo.^270^ Negatively charged NPs tend to have a higher degree of stability in biological fluids and can be taken up more efficiently by cells.^271^ They also tend to interact less with proteins, which can reduce the risk of an immune response. On the other hand, positively charged NPs can have a greater tendency to aggregate and can be rapidly cleared by the RES, which can limit their circulation time in the body.^272^ The charge of NPs can also affect their biodistribution patterns. Positively charged NPs tend to accumulate in the liver and spleen, whereas negatively charged NPs can have a more widespread distribution throughout the body.^273^ Another important factor is the charge density of the NPs, which can directly affect the behavior and stability of the NPs in in vivo. High charge density can improve the stability of the NPs and can also enhance the toxicity along with rapid clearance.^274^ The charge of NPs is an important consideration in the design and development of NPs for in vivo applications, as it can have a significant impact on biodistribution patterns, their stability, and circulation time.^275^

## 8 CURRENT LIMITATIONS

NMs have the potential that can be implemented in cancer diagnostics and therapies.^276^ However, it is critical to address the opposite side of the coin, specifically, unexpected harmful effects of NMs like cytotoxicity, effect of size on toxicity, retention period, biodistribution, efficacy, and physiological response, which have been widely studied.^277^ Many of them appear to conflict with one another. The lack of consistent knowledge on NMs could cause delirium and have a severe influence on human health.^278^ Although the limitations mentioned in general are applicable toward any NP, the following instances are specific to AuNPs. Toxicity: AuNPs toxicity to biological systems has long been a subject of research. Surface chemistry, shape, targeted ligand, size, elasticity, and composition of AuNPs all have an impact on their toxicity.^279^ Together with the complexity and variability of human cells and tissues creates a challenging situation and makes it difficult to investigate the effect and responses of the biological system to the delivery of AuNPs in a comprehensive manner.^280^ The toxicity of AuNPs has been linked to their surface charge. It was identified that positively charged particles are more harmful than negative or neutral.

NPs are the basic building blocks for the several important applications in the scientific fields because of their distinct characteristics, such as targeting potential, high surface‐to‐mass ratio, and capability of adsorbing and transporting additional compounds. This qualifies them for biomedical application.^281^ Application of NMs and innovative nanotechnological development can be a successful potential strategy. NMs improve present technological efficiency, but they can also enhance the amount of water that can be used. On the other hand, NMs will assist in the establishment of technologically advanced and highly efficient treatment facilities.^282^ This will be helpful to lower the cost of anticancer drugs and medication development. To meet these objectives, NMs need to be produced at a low cost while maintaining large synthesis. Reusability, stability, and workability in industrial applications are other important considerations for their improvement and large‐scale production.^283^ The toxicity of NMs in vitro and in vivo must be evaluated in both short‐ and long‐term approaches. Despite the fact that nanotechnology is the most recent industrial development, there is certainly a requirement for strong and well standards to be established regarding the application, release, and processing requirements for NMs.^5^

## 9 CONCLUSION AND FUTURE PERSPECTIVES

The greatest problems in cancer therapy have emerged with the advancement of modern healthcare. The theranostic approach, which is a combination of therapy and diagnosis, is gaining popularity. It has been proposed as a technique for assisting the transition from traditional, nonspecific medicine to modern precision or personalized treatment. Compared with conventional screening modalities, for example, radionuclide and MRI, optical imaging has several distinct benefits that make it a desirable diagnostic tool. Transition metal complexes have recently gotten a lot of focus as a potential substitute to traditional dyes in optical sensors because of their high stokes shifts, extended lives, and simplicity of structural modification. Because of the inherent disadvantages of optical therapy, clinical and preclinical research studies demonstrated that PTT alone is often difficult to destroy malignancies and kill deep‐located cancers. To improve PTT's therapeutic benefit while reducing its substantial adverse effects, a new direction combining PTT with other treatment techniques is desired. Due to their unique physicochemical features, two‐dimensional TMDCs, as typical ultrathin 2D layer NMs, have recently attracted attention in a variety of sectors, including biomedicine.

Therefore, the development of effective and safe chemotherapeutic agents is important, to strengthen the quality of life, boost the rate of survival, and, most significantly, and extending the lifespan of humans. NM‐assisted combination medicines are becoming more prevalent as nanoscience progresses. Metal‐based NPs recommended resolving the drug resistance. These NPs interact positively with biomolecules in the cell and can be more effectively used in the development of potential medications for the treatment of cancer. Metallic‐based NPs have been suggested to defeat cancer and to increase the effectiveness of healing, diagnosis, photoacoustic imaging, PTT, valuable for prophylactic, antiviral medications, and laser therapy for cancer cells. Because of their antiviral, antibacterial, anti‐inflammatory, and antifungal properties, NPs have a lot of potentials to be used as anticancer agents. The molecular mechanisms and relationships that support these kinds of characteristics are not completely explored. The relationships of NMs with cellular and biotic ecological systems, especially in the modification of NPs, are another topic that requires further investigation. Finally, there is an essential need to investigate the toxicological and pharmacokinetics features of metal‐based complexes; these NPs are the most innovative and have potential in the area of chemotherapeutics. In this review, the current developments, the recent potential of the plant and metal‐based NMs, and current limits are discussed, as well as recommendations for more effective use of NMs in cancer therapy.

## AUTHOR CONTRIBUTIONS

*Literature review; writing—original draft preparation and figures*: Sunil Kumar. *Editing*: Abhishek Kumar Sharma, Monu Kumar Shukla, and Gururaj K. Jayaprakash. Rajiv K. *Review*: Tonk and Dinesh K. Chellappan. *Conceptualization*: Sachin Kumar Singh, Kamal Dua, and Faheem Ahmed. *Supervision: Sanjib Bhattacharyya and Deepak Kumar*. All authors read and approved the final manuscript.

## CONFLICT OF INTEREST STATEMENT

The authors declared no potential conflict of interests with respect to the research, authorship, and/or publication of this article.

## ETHICS STATEMENT

Not Applicable.

## Data Availability

Not Applicable.
